# Effect of BMP-2 Delivery Mode on Osteogenic Differentiation of Stem Cells

**DOI:** 10.1155/2017/7859184

**Published:** 2017-01-19

**Authors:** Taekhee Jung, John Hwan Lee, Soonjung Park, Yong-Jin Kim, Joseph Seo, Hye-Eun Shim, Ki-Suk Kim, Hyon-Seok Jang, Hyung-Min Chung, Seong-Geun Oh, Sung-Hwan Moon, Sun-Woong Kang

**Affiliations:** ^1^Department of Stem Cell Biology, School of Medicine, Konkuk University, Seoul, Republic of Korea; ^2^Department of Chemical Engineering, Hanyang University, Seoul, Republic of Korea; ^3^AmorePacific Corp./R&D Center, Yongin-si, Gyeonggi-do, Republic of Korea; ^4^Predictive Model Research Center, Korea Institute of Toxicology, Daejeon, Republic of Korea; ^5^Department of Human and Environmental Toxicology, University of Science and Technology, Daejeon, Republic of Korea; ^6^Department of Dentistry, Korea University, Ansan Hospital, Ansan, Republic of Korea

## Abstract

Differentiation of stem cells is an important strategy for regeneration of defective tissue in stem cell therapy. Bone morphogenetic protein-2 (BMP-2) is a well-known osteogenic differentiation factor that stimulates stem cell signaling pathways by activating transmembrane type I and type II receptors. However, BMPs have a very short half-life and may rapidly lose their bioactivity. Thus, a BMP delivery system is required to take advantage of an osteoinductive effect for osteogenic differentiation. Previously, BMP delivery has been designed and evaluated for osteogenic differentiation, focusing on carriers and sustained release system for delivery of BMPs. The effect of the delivery mode in cell culture plate on osteogenic differentiation potential was not evaluated. Herein, to investigate the effect of delivery mode on osteogenic differentiation of BM-MSCs in this study, we fabricated bottom-up release and top-down release systems for culture plate delivery of BMP-2. And also, we selected Arg-Gly-Asp- (RGD-) conjugated alginate hydrogel for BMP-2 delivery because alginate is able to release BMP-2 in a sustained manner and it is a biocompatible material. After 7 days of culture, the bottom-up release system in culture plate significantly stimulated alkaline phosphate activity of human bone marrow-mesenchymal stem cells. The present study highlights the potential value of the tool in stem cell therapy.

## 1. Introduction

An in vitro differentiation process to obtain specific cell type from stem cells is required for stem cell therapy. Stem cells can be manipulated in vitro under specific conditions that favor differentiation towards a designated somatic cell type [1]. Many studies have demonstrated manipulative techniques to direct stem cell differentiation through use of defined media, substrates and growth factors [2]. In particular, bone morphogenetic protein-2 (BMP-2) is a well-known inductive growth factor for osteogenic differentiation of various stem cells [3]. BMP-2 binds to microdomains on the cellular surface related to biological signal pathways, such as cognate receptors, to induce osteogenic differentiation [4]. Thus, the probability of BMP-2 binding to surface receptors should be maximized to enhance efficacy of osteogenic differentiation during BMP-2 treatment process in vitro.

Protein delivery system is a promising method for localized and sustained delivery of biologically active BMP-2 at the target sites [5]. Conventional methods involve the daily addition of BMP-2 to the culture medium and BMP-2 is assumed to be homogeneous as well as sufficient in the medium [6]. However, only a small amount reaches the cellular microdomains related to the biological signal pathways because of Brownian motion of BMP-2 in the culture medium. In contrast, BMP-2 released from a matrix utilizing a protein delivery system could efficiently bind to receptors on the cultured cells. However, such delivery systems rarely focus on monolayer cultures subject to conventional techniques. Previous studies have not compared the effect of BMP-2 delivery modes on stem cells in a monolayer culture system that provides convenience and speed to obtain a large number of desired cells, such as osteocytes.

The purpose of this study was to investigate the effects of BMP-2 delivery mode on the osteogenic differentiation of human bone marrow-derived mesenchymal stem cells (BM-MSCs). To that end, BMP-2 was loaded to Arg-Gly-Asp (RGD) peptide-conjugated alginate hydrogel. We selected alginate as a base material for BMP-2 delivery in this study because alginate has valuable properties such as biocompatibility and gel-forming properties via ionic crosslinking using calcium in mild condition. In addition, this reaction is rapid and selective and produces high yields. Thus, this can be used as a carrier of BMP-2 and to create a suitable environment for cell culture. Human BM-MSCs were induced by using a bottom-up and top-down release system and the cells were characterized in terms of alkaline phosphatase (ALP) activity and differentiation. The results may provide a useful tool for expanding the potential applications of stem cell therapy.

## 2. Methods and Materials

### 2.1. Synthesis of Peptide-Modified Alginate

Sodium alginate (*M*_*w*_ = 200,000–300,000; FMC Biopolymer, Philadelphia, PA) was dissolved in a 2-(*N*-morpholino)ethanesulfonic acid (MES) buffer at room temperature (pH = 6.5, 0.3 M NaCl). A peptide with the (glycine)_4_-arginine-glycine-aspartic acid-alanine-(serine)_2_-lysine (G4RGDASSK) sequence (Anygen, Seoul, Republic of Korea) was added to the alginate solution in the presence of* N*-hydroxysulfosuccinimide (sulfo-NHS; Pierce, Rockford, IL) and 1-ethyl-3-(dimethylaminopropyl)carbodiimide (EDC, Sigma-Aldrich, St. Louis, MO). The peptide-modified alginate was purified by extensive dialysis with distilled water for 5 days (*M*_*w*_ cut-off = 3,500) and activated charcoal treatment and then sterilized with a 0.22 *μ*m filter. The degree of substitution (DS) of the peptide was determined with the number of peptides per 100 uronic acid residues in the alginate chain. In this study, the DS was 0.15 [7].

### 2.2. Preparation of Hydrogels and Encapsulation of BMP-2

The purified and lyophilized RGD-modified alginate (60 mg) was dissolved in*α*-MEM (3 mL) and mixed with the Chinese Hamster Ovary (CHO) cell-derived recombinant human BMP-2 (R&D Systems, Minneapolis, MN). A calcium sulfate (CaSO_4_) solution (20% w/v, 120 *μ*L) was added to a second syringe. The two syringes were connected with a female connector, and the contents were quickly mixed. An alginate solution containing CaSO_4_ formed a gel at 37°C for 20 min. The gel was used to punch out discs (8 mm diameter; 0.5, 1, and 2 mm thickness) used for release test of BMP-2 and for culture of cells.

### 2.3. Determination of the Kinetics of BMP-2 Release from RGD-Alginate Hydrogel Discs

To determine the degree of BMP-2 release from RGD-alginate hydrogel discs (2% w/v) with 0.5, 1, and 2 mm thickness, BMP-2-loaded scaffolds were sunk in 24-well culture plates containing 1 mL phosphate-buffered saline (PBS, pH 7.4; Sigma) and the culture plates were incubated at 37°C without agitation. At predetermined times, enzyme-linked immunosorbent assay (ELISA) was done to determine the kinetics of BMP-2 release from RGD-alginate hydrogel discs with various thicknesses. At each time point, supernatant was collected and the culture plates were replenished with fresh buffer. The amount of BMP-2 in the supernatants was measured using an ELISA kit (human beta BMP-2 Duoset; R&D Systems). Briefly, ELISA plates (NUNC, Polylabo, Strasbourg, France) were coated with the capture monoclonal antibody and then blocked with the bovine serum albumin (1 w/v%) and sucrose (5 w/v%) for 1 hour. After appropriately diluted supernatants were added, bound-BMP-2 was detected with biotin-conjugated anti-human BMP-2 polyclonal antibody. Streptavidin-conjugated horseradish peroxidase was then added to the plates. Enzyme substrate (tetramethylbenzidine and peroxide) was treated for 20 minutes, and the reaction was stopped by adding an acidic solution. Absorbance was measured at 450 nm range of PowerWave X340 plate reader (Bio-TEK Instrument, Inc., Winooski, VT). The amount of BMP-2 was calculated from a calibration curve based on known concentrations of BMP-2. Experiments were performed with five replicates of each supernatant.

### 2.4. Cell Culture and Differentiation

Human BM-MSCs were purchased (Lonza Ltd., Walkersville, MD) and cultured in accordance with a previously described method [8]. Briefly, cells were maintained in *α*-MEM (Gibco BRL, Grand Island, NY) supplemented with 10% (v/v) fetal bovine serum (FBS; Gibco BRL), 2 mM L-glutamine (Gibco BRL), 100 units/mL penicillin (Gibco BRL), and 0.1 mg/mL streptomycin (Gibco BRL). In this study, BM-MSCs were used after three to five passages. The stem cell markers (CD 44 (95.39%), CD 73 (93.13%), CD 90 (98.60%), and CD 105 (94.71%)) for BM-MSCs were analyzed by fluorescence activated cell sorting (FACS) analysis which characterized BM-MSCs at passage 5 by flow cytometry histograms (see Supplementary Figure 1 in Supplementary Material available online at https://doi.org/10.1155/2017/7859184).

To determine RGD modification of alginate, the BM-MSCs were seeded onto the surfaces of alginate hydrogel discs at density of 2 × 10^4^ cells/cm^2^. The discs were placed in 24-well culture plates and incubated at 37°C under 5% CO_2_ atmosphere. After a 24 h culture period, discs were washed with PBS to remove nonadhered cells, and then photographs of the BM-MSCs adhering to the surface of the discs were taken using an optical microscope (Olympus, Tokyo, Japan). For the osteogenic differentiation of BM-MSCs, the BM-MSCs and RGD-modified alginate hydrogel discs containing BMP-2 (2 *μ*g/disc) were applied as shown in Figures 2 and 3. Briefly, the BM-MSCs were seeded onto RGD-modified alginate hydrogel discs with BMP-2 and cultured with*α*-MEM for 7 days to obtain the bottom-up release system. To construct the top-down release system, BM-MSCs were seeded onto RGD-modified alginate hydrogel discs without BMP-2 and RGD-modified alginate hydrogel discs with BMP-2 were placed in Transwell inserts. Following the adhesion of the BM-MSCs, the cells were cultured with Transwell inserts containing RGD-modified alginate hydrogel discs with BMP-2 in *α*-MEM for 7 days, with the culture media changed every other day.

### 2.5. ALP Assay

To investigate the effects of BMP-2 delivery mode on BM-MSCs osteogenic differentiation, ALP activity as an early osteogenic differentiation marker was measured after 7 days, when cells on alginate hydrogel discs were stained using an ALP staining kit II (Stemgent, Lexington, MA) according to the manufacturer's instructions. The cells on each alginate gel disc were observed and photographed with an optical microscope (Nikon, Tokyo, Japan). In addition, the cells were lysed to quantify ALP activity as described previously [9]. ALP activity was normalized by the protein content, which was examined using the BCA protein assay reagent (Pierce Chemical, Rockford, IL). The BM-MSCs cultured on RGD-modified alginate without BMP-2 were used as negative control (Supplementary Figure 2).

### 2.6. Statistical Analysis

The quantitative data are expressed as means ± standard deviations (SD). Statistical analyses were performed by one-way analysis of variance (ANOVA) with Statistical Package for the Social Sciences (SPSS) software (SPSS Inc., Chicago, IL). A value of *p* < 0.05 was considered statistically significant.

## 3. Results

### 3.1. Preparation of RGD-Modified Alginate Hydrogel for Human BM-MSC Culture

**Figure 1:**
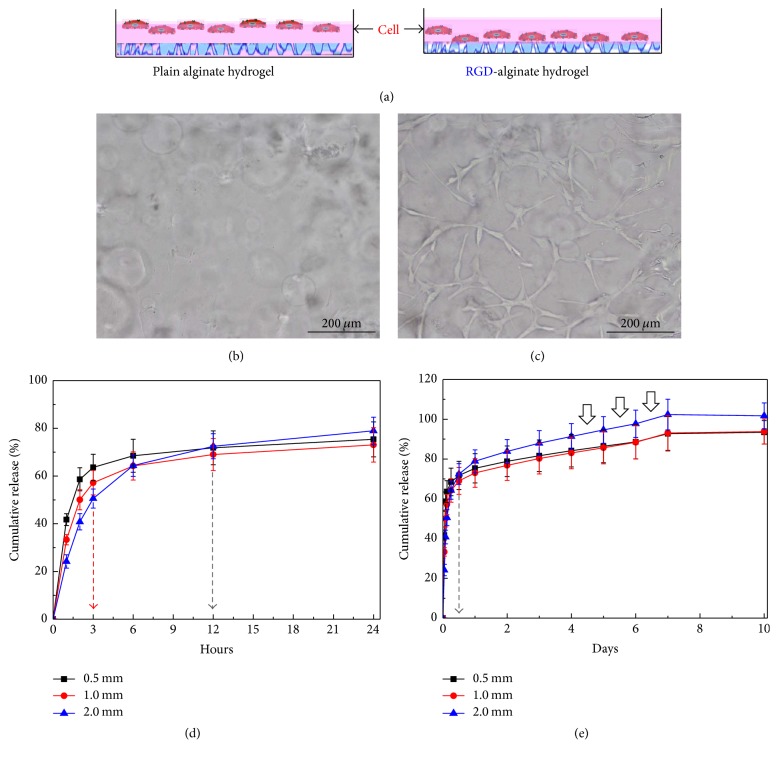
Role of RGD peptide in cell adhesion during culture of BM-MSCs. (a) Experimental design for plain and modified RGD-alginate hydrogel. Photographs of human BM-MSCs adhered on the surface of (b) unmodified alginate hydrogel disc and (c) RGD-modified alginate hydrogel disc 1 day after cell plating. The profiles of BMP-2 release from RGD-modified alginate hydrogel disc with various thicknesses (0.5, 1.0, and 2.0 mm) (d) for 24 hours and (e) 10 days. The amount of BMP-2 released from various hydrogel discs was determined by ELISA. The values represent the mean ± standard deviation (*n* = 5).

We first performed chemical conjugation of RGD sequence containing GGGGRGDASSK peptide into the alginate hydrogel. Because the attachment capacity of cells is low on alginate hydrogel, it is ideal for the investigation of RGD influence on adhesion of cells. Applicability was examined by plating cells (2 × 10^4^ cells/cm^2^) on an unmodified (control) and RGD-modified alginate hydrogel disc (Figure 1(a)). After 24 hours of culture, the unmodified group showed that the cells remained in suspension and were unable to adhere (Figure 1(b)). In contrast, cell adhesion in the RGD-modified group was favorable and the attached cells exhibited a fibroblastic morphology (Figure 1(c)). This result indicated that the peptide containing RGD was successfully conjugated to the alginate backbone, resulting in enhancements of cell adhesion on hydrogel disc that does not naturally possess adhesive properties.

### 3.2. Analysis of the Kinetics of BMP-2 Release from RGD-Alginate Hydrogel Discs with Various Thicknesses

Next, we loaded BMP-2 (2 *μ*g/disc) into RGD-modified alginate hydrogel discs to determine the optimal thickness for releasing BMP-2 effectively into the cell culture plate. The RGD-modified alginate hydrogel discs with various thicknesses (0.5 mm, 1.0 mm, and 2.0 mm) were fabricated and placed in cell culture plates to test the kinetics of BMP-2 release from alginate hydrogel discs. The release of BMP-2 from 0.5 mm thickness-alginate discs was more rapid than that from 2.0 mm thickness-alginate discs (Figure 1(d)). Almost all of the BMP-2 was released from the alginate hydrogel discs within the first 10 days (Figure 1(e)). Importantly, the release of BMP-2 from 2.0 mm thickness-alginate hydrogel discs was sustained for 12 hours. The BMP-2 release rate decreased as the thickness of alginate hydrogel discs increased. In addition, with the 0.5 mm discs, approximately 90% of the initially loaded BMP-2 was released over the first 10 days. In contrast, for the 2.0 mm discs, virtually all of the loaded BMP-2 was released over the first 10 days. The present findings indicate that the rate of BMP-2 release from alginate hydrogel disc can be controlled by the thickness of hydrogel disc. A disc thickness of 2.0 mm provides favorable kinetics of BMP-2 release for osteogenic differentiation of stem cells.

### 3.3. Osteogenic Differentiation of BM-MSCs in Top-Down and Bottom-Up Release Systems for BMP-2 Delivery

**Figure 2:**
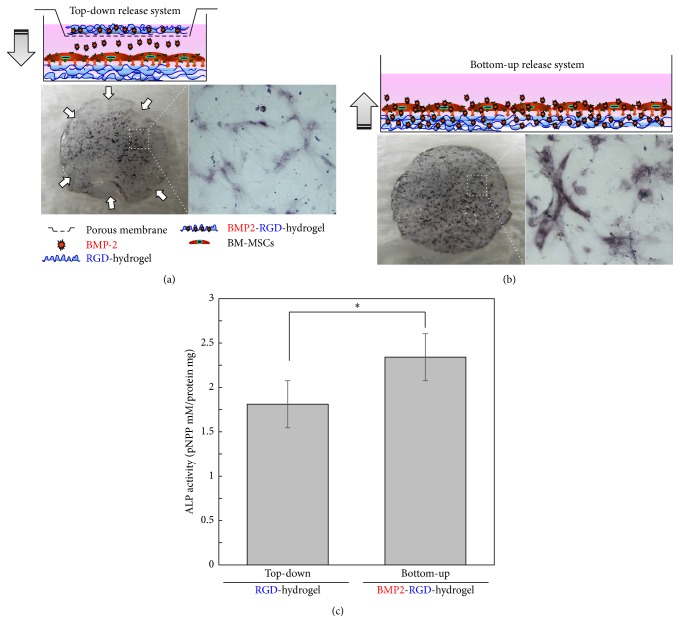
Schematic representation of culture system on alginate hydrogel disc used to assess the effect of BMP-2 delivery mode on osteogenic differentiation of human BM-MSCs and photographs of ALP stained human BM-MSCs. (a) Top-down release system, (b) bottom-up release system, and (c) quantification of ALP activity for BM-MSCs cultured under each mode of delivery. The values represent the mean ± standard deviation (*n* = 3). ^*∗*^*p* < 0.05 compared with top-down release system at 7 days.

In order to investigate whether BM-MSCs differentiation was influenced by the mode of BMP-2 delivery, BM-MSCs were cultured in a top-down or bottom-up release system (Figure 2). Similar to standard protocols, the top-down approach was structured to release BMP-2 into the media through an 8 *μ*m porous membrane. In contrast, the bottom-up system was set up in order to make the RGD-alginate hydrogel be in contact with BM-MSCs during BMP-2 release. This approach utilized adhesion and proximity in order to force the cells to be more interactive with the protein. The osteogenic differentiation of BM-MSCs was analyzed using ALP staining and ALP activity (mM/protein mg). Results of ALP staining showed that BM-MSCs cultured in the bottom-up release system were intensively stained compared to those in the top-down release system (Figures 2(a) and 2(b)), demonstrating that BM-MSC differentiation into osteogenesis occurred better in the bottom-up release system than the top-down release system. Additionally, we analyzed ALP activity (mM/protein mg) in both release systems. The bottom-up release system produced increased protein level in comparison to the top-down release system (Figure 2(c)), confirming that differentiation was more favorable in the bottom-up release system.

**Figure 3:**
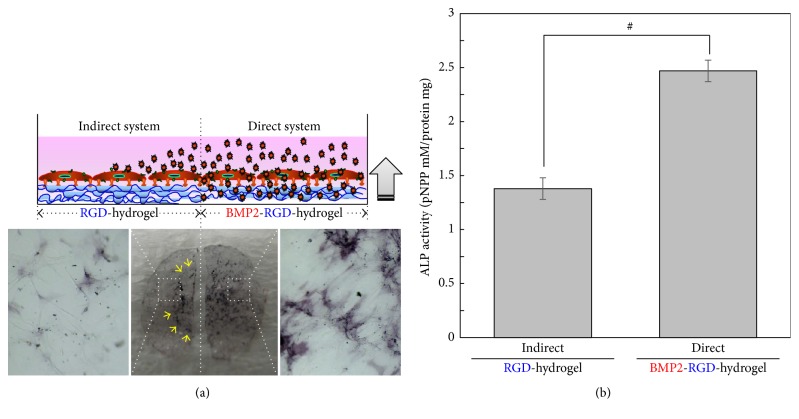
A top-down and bottom-up approach applied to a one culture dish. (a) ALP staining and (b) quantification of ALP activity for BM-MSCs cultured in their respective half. The values represent the mean ± standard deviation (*n* = 3). ^#^*p* < 0.05 compared with direct bottom-up release system at 7 days.

Based on these results, we confirmed that differentiation of stem cells was influenced by the method of release. To further investigate this, BM-MSCs in the same culture discs were divided into two sections consisting of an untreated RGD-hydrogel and modified BMP2-RGD-hydrogel. As expected, ALP stains appeared denser in the half of the discs receiving BMP-2 in a bottom-up manner (Figure 3(a)). However, it is very likely that BMP-2 released from the bottom-up system indirectly carried over to the untreated section, which may have influenced adjacent cells to exhibit signs of differentiation (Figure 3(a), yellow arrow). In addition, measured ALP activity remained higher in the direct-contact group regardless of disc division (Figure 3(b)).

## 4. Discussion

We investigated the effect of the delivery mode of BMP-2 on osteogenic differentiation of human BM-MSCs. The top-down release system and bottom-up release system were used to investigate the effects of release mode of BMP-2. The main difference of both systems is how the BMP-2 was transmitted to the cells.

Compared to conventional method for BMP-2 treatment in two-dimensional cell culture, the bottom-up release system presents several advantages. First, BMP-2 appeared to bind more rapidly and readily to receptors on cultured stem cells than BMP-2 released from top-down release system. In top-down release system (conventional method), BMP-2 released in the culture medium showed Brownian motion [10]. Thus, only a small amount of BMP-2 reaches the receptors related to osteogenic differentiation pathway on the cells. In contrast, BMP-2 released from bottom-up system may trigger cell signals more efficiently. Although the release profiles of BMP-2 in both systems are equal, the osteogenic differentiations of stem cells cultured on bottom-up release system were superior to those of cultures with top-down release system (Figure 2). This can be explained by the rapid binding of BMP-2 released from the alginate hydrogel to receptors on human BM-MSCs. Second, alginate gel as a BMP-2 reservoir may play an important role at the delivery site to ensure their proper biological activity. In this study, half of cell culture plate is coated with alginate hydrogel containing BMP-2 (direct system) and half of cell culture plate is coated with only alginate hydrogel without BMP-2 (indirect system) (Figure 3). The BMP-2 released from alginate hydrogel in the direct system rapidly triggered osteogenic differentiation of stem cells. The direct system induced ALP activity to a much greater extent than that of indirect system. In contrast, the osteogenic differentiation of stem cells was interrupted at indirect culture sites compared to that of direct system. This indicates that delivery mode of BMP-2 may also influence the efficacy of osteogenic differentiation.

In this study, daily addition of BMP-2 was not used as a control group. Several studies have reported successful osteogenic differentiation using various BMP-2 release systems [11, 12]. In these studies, cells were cultured in culture plates with BMP-2 added to the culture medium daily as control group for evaluation of BMP-2 release system. The daily addition of BMP-2 is based on the assumption that the total concentration of BMP-2 remains constant. However, actual amount of BMP-2 in culture media is different as indicated by BMP-2 release profile. Therefore, conventional control may be inefficient to test effect on osteogenic differentiation by delivery mode of BMP-2. Instead, we deliberately used control groups with equal release profile (top-down release system) to avoid complicating systemic factors for a more equal comparison of the two types of BMP-2 delivery mode.

**Figure 4:**
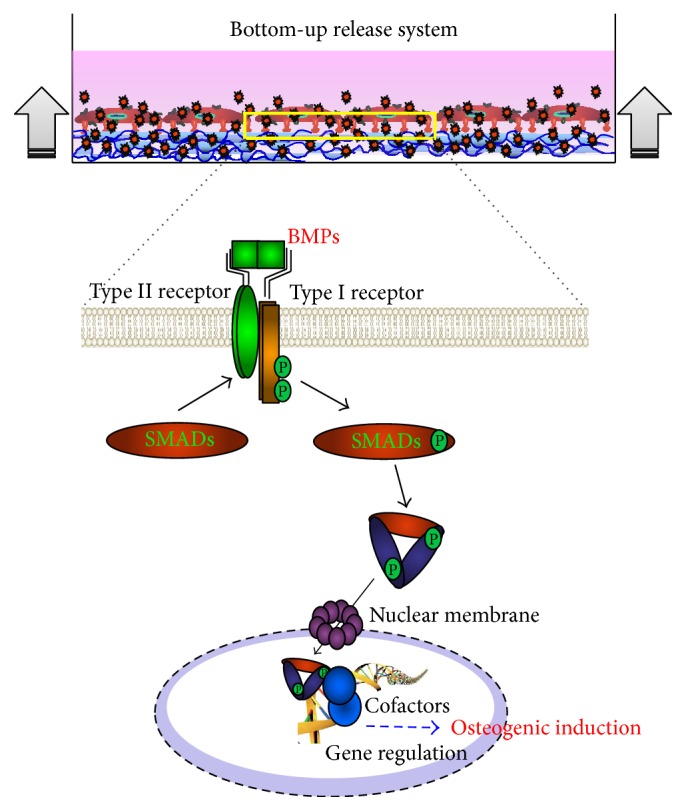
Schema of osteogenic differentiation through the BMP-2 signaling pathway. Type I and type II BMP receptors span the cell membrane and bind extracellular BMP ligand. Ligand binding to BMP receptor complexes activates signaling through type II-receptor-mediated phosphorylation of the type I receptor. In bottom-up release system, the probability of BMP-2 binding to receptors was maximized during BMP-2 treatment process.

Depending on the type of cell, BMP-2 is involved in the hedgehog pathway, transforming growth factor-beta signaling pathway, and cytokine-cytokine receptor interaction [13, 14]. Previous studies have reported its involvement in extra-embryonic endoderm derivation from human embryonic stem cells and chondrogenic commitment of MSCs as well as cardiomyocyte contractility [15–17]. While the action of BMP-2 is relatively extensive, in mesenchymal stromal cells, it serves as an important factor for osteogenic commitment in a ligand-dependent manner to activate downstream gene regulation via SMAD [18] (Figure 4). Thus, we believe that the enhancement of the osteogenic differentiation was caused by the increased osteogenic activity of BM-MSCs that was presumably triggered by the enhancement of BMP-2 binding in bottom-up release system. Future studies are needed to elucidate the molecular mechanisms of BMP-2 binding effects in the bottom-up release system.

In conclusion, osteogenic differentiation of BM-MSCs was significantly enhanced in the bottom-up release system compared to those of the top-down release system. These results show that the bottom-up release system could serve as a differentiation stimulator of stem cells. Thus, these findings could be useful for applications involving stem cell culture or differentiation studies that aim to advance cell utility in the field of stem cell therapy.

## Supplementary Material

In Supplementary Figure 1, BM-MSCs were cultivated up to five passages and we subsequently examined representative MSC markers by FACS analysis. MSC specific markers, CD 44 (95.39%), CD 73 (93.13%), CD 90 (98.60%), and CD 105 (94.71%), were strongly expressed in the cultured population. This result showed that the differentiated cells expressed relevant MSC markers which indicate that the cells possessed MSC characteristics. In Supplementary Figure 2, since MSCs induced towards an osteogenic fate are known to strongly express ALP, BCA protein assay reagent was used to normalize ALP activity by the protein content for examination. BM-MSCs cultured on RGD-modified alginate without BMP-2 were used as negative control.
