# Supplementary material for: Effect of BMP-2 Delivery Mode on Osteogenic Differentiation of Stem Cells
Source: Stem Cells Int. 2017 Jan 19;2017:7859184. doi: 10.1155/2017/7859184 (PMC5288534; doi:10.1155/2017/7859184)
Supplement: Supplementary file 1 — In Supplementary Figure 1, BM-MSCs were cultivated up to five passages and we subsequently examined representative MSC markers by FACS analysis. MSC specific markers, CD 44 (95.39%), CD 73 (93.13%), CD 90 (98.60%), and CD 105 (94.71%), were strongly expressed in the cultured population. This result showed that the differentiated cells expressed relevant MSC markers which indicate that the cells possessed MSC characteristics. In Supplementary Figure 2, since MSCs induced towards an osteogenic fate are known to strongly express ALP, BCA protein assay reagent was used to normalize ALP activity by the protein content for examination. BM-MSCs cultured on RGD-modified alginate without BMP-2 were used as negative control. [file 7859184.f1.pdf]

Supplementary Figure 1

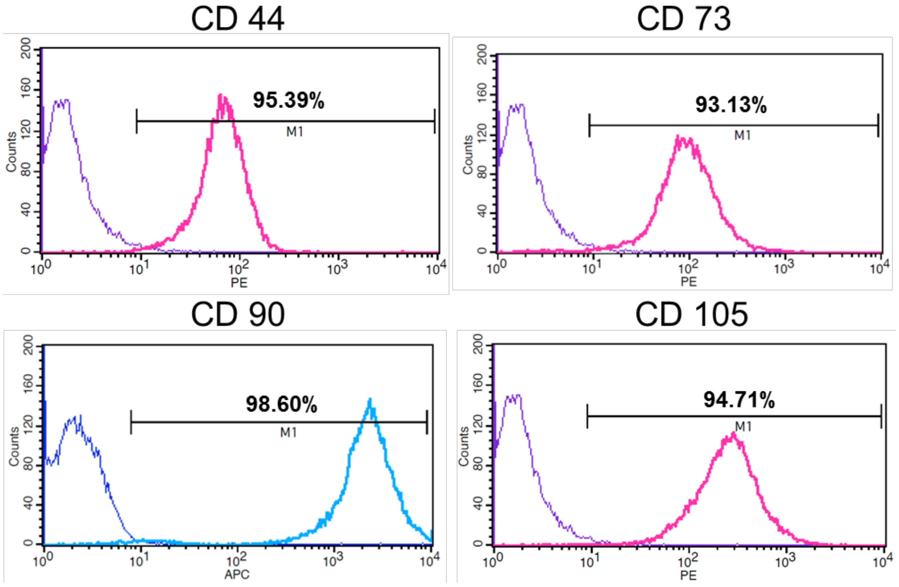

Supplementary Figure 1. Characterization of BM-MSCs at passage 5 by flow cytometry histograms.

Supplementary Figure 2

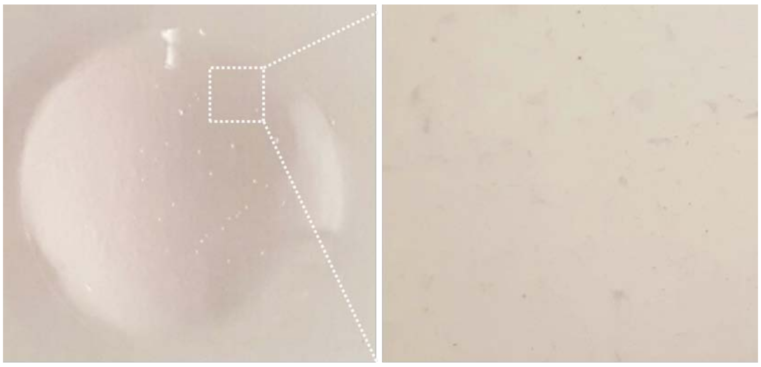

Supplementary Figure 2. The photographs of an ALP stained human BM-MSCs cultured on RGD-modified alginate without BMP-2.
